# Recovery services and expectation of consumers and mental health professionals in community-based residential facilities of Ghana

**DOI:** 10.1186/s12888-020-02768-w

**Published:** 2020-07-06

**Authors:** Naomi Gyamfi, Eric Badu, Wisdom Kwadwo Mprah, Isaac Mensah

**Affiliations:** 1 Department of Health Promotion and Disability Studies, Kwame Nkrumah University of Science and Technology, Kumasi, Ghana; 2 School of Health, University of New England, Armidale, Australia; 3 School of Nursing and Midwifery, University of Newcastle, Newcastle, Australia; 4 Department of Special Education, University of Education, Winneba, Ghana

**Keywords:** Community-based mental health, Expectations, Ghana, Personal recovery journey, Recovery services, Rehabilitation

## Abstract

**Background:**

In the past decades, considerable global attention has been drawn to recovery services that seek to promote the personal recovery journey of consumers with mental illness. However, in most settings, including Ghana, limited empirical studies have attempted to explore, from the perspectives of Mental Health Professionals (MHPs) and consumers, the effectiveness of recovery services and expectation towards the recovery. This study, therefore, explored consumers’ and MHPs perspectives concerning recovery services and expectations towards recovery in two community-based residential facilities in Ghana.

**Methods:**

A qualitative method, involving in-depth interviews and observations, were used to collect data from 24 participants (5 MHPs and 19 consumers). Thematic analysis was used to analyze the data.

**Results:**

The study identified three global themes and nine organizing themes. The global themes were recovery services offered to consumers, expectation regarding personal recovery and challenges in achieving recovery. The study found that recovery services were expected to improve the internal and external recovery processes of consumers. The internal recovery process was independent living whilst the external recovery process were management of illness, economic empowerment and social inclusion. Several systemic and consumer-related factors influenced consumers’ and MHPs expectation concerning the recovery journey.

**Conclusion:**

The study concludes that the government should prioritize the use of recovery services through policies, financial incentives, infrastructure support, and adequate training of MHPs.

## Background

Globally, mental health practitioners and consumer groups are advocating for governments to support the use of recovery services because these services ensure effective recovery of mental health consumers [1–5]. Recovery services promote effective restoration of functioning, mental well-being, and quality of life of consumers through a holistic approach [6, 7]. The approach focuses on the strengths of an individual rather than the illness [8]. The recovery practices are supported by a collaborative partnership among consumers, carers, and Mental Health Professionals (MHPs) [2, 4]. Such practices also place consumers first and at the centre of care. In addition, they promote culture and language of hope and take actions that promote social inclusion [1, 3, 9–17]. For example, the 1990’s conceptualization of recovery explained it as a deeply personal, unique process of changing one’s goals, skills, roles, attitudes, feelings and values, thus, living a satisfying, hopeful, and contributing life even with limitations caused by mental illness [18].

The recovery process is classified into clinical and personal [19, 20]. The clinical recovery involves the remission of symptoms, that is, achieving the long-term goal of psychosocial functioning with fewer or no relapses [19, 20]. The personal recovery highlights the consumer-based or subjective perspectives [19, 20]. The personal recovery is further categorized into internal and external [10, 12, 16, 21]. The internal process of recovery describes the inner strength and willpower of learning about one’s self, understanding the illness, changing one’s lifestyle and reclaiming various aspects of self [10, 12, 21]. Recovery, as an external process describes consumers of mental health services as active and productive members of society, who are living well despite the symptoms of mental illness [10, 12]. Here, consumers aim to attain an optimum level of functioning [2, 10, 22], normative life as well as connectedness [1, 9, 14, 16, 17, 19]. Achieving recovery requires that stakeholders should consider the social context and cultural systems that define healthy and fulfilling life and goals [23].

Worldwide, services that are implemented as part of the recovery process are categorized into integrated rehabilitation treatment [24–30]; vocational rehabilitation [31–38]; and recovery narrative photovoice, art-making, and exhibition services [39–43]. Integrated rehabilitation treatment involves a collaborative and integrated approach to treatment (for example. Illness management, mindfulness-based therapy, cognitive behavioural therapy). The vocational rehabilitation services (eg. individual placement and support, sheltered employment, supported employment, and social enterprises) are evidence-based interventions, which assist consumers to gain competitive employment. It is an evidence-based approach that supports consumers in their efforts to achieve steady, meaningful employment in mainstream competitive jobs, as either part-time or full-time employees. This involves job search, follow-up support, and counselling services in a community setting [31, 32, 34, 35, 38]. Recovery narrative photovoice, art-making, and exhibition services involve the use of photographs and text to construct consumers’ experiences regarding mental illness. With this, consumers use photos and text to describe their expectation regarding recovery [39, 41, 42].

In recent times, assessment of consumers’ and MHPs expectation regarding mental health services is considered relevant in building partnership between these stakeholders and in developing their shared goals [16, 17, 44]. For example, listening to consumers’ expectations, and what they hope to achieve, helps to examine their priorities and preferences [17, 44]. Thus, empirical evidence is needed to explore consumers’ and MHPs’ expectation concerning recovery services. Despite this, research evidence on such expectations of consumers and MHPs is limited. The few evidence regarding consumers’ and MHPs’ perspectives on recovery and expectation are conducted in developed countries, such as Australia, UK and US [16, 17, 44].

In developing countries, there is limited evidence in terms of understanding of and effectiveness of recovery-based approaches. For example, in Ghana, no empirical study has been conducted to explore the consumers’ and MHPs’ expectation towards recovery, particularly in a community-based residential setting. Empirical studies on mental health services in Ghana are largely limited to governance and policy, systems strengthening [45–47], treatment pathways [48–50] and burden of family caregiving [51–53]. Therefore, this paper aims to contribute to this gap by answering the following questions 1) what are MHPs’ and consumers’ perspectives concerning the recovery services, and 2) what are the expectation of consumers and MHPs towards the recovery journey.

## Methods

### Study setting

The study was conducted in two community-based residential mental facilities in the Kumasi Metropolis and Ejisu-Juaben Municipality, both in the Ashanti region of Ghana [54]. The two facilities are among the three community-based residential facilities in the Ashanti region of Ghana [55]. The two facilities were purposively selected because they operate a shared model of service delivery, which aim to reintegrate adult consumers of mental health services into their communities. Table 1 illustrate the characteristics of the services (eg. strength and capacity) of the residential facilities.

**Table 1:** Total staff, consumers and type of interventions provided at the residential facilities, adopted from Parker, Dark [17]

Indicators	Description of profession	Facility 1	Facility 2
Facility	Location	Kumasi Metropolis	Ejisu-Juaben District
	The minimum duration of rehabilitation	9–18 months	–
MHPs	Total FTE staff	13	6
	Total FTE clinical staff	2	3
	Non-Clinical staff	11	3
	Rotational (Casual nurses)	Min 10 max 30	Min 10 max 30
	Total peer-support staff	5	3
	Consumer: FTE staff ratio	3:1	9:1
Consumers	Maximum occupancy (consumers)	40	55
Physical Environment	Number of self-contained independent living units	None	None
	Number of shared halls and rooms for living	22	6
	Total number of beds for consumers	55	–
Philosophy of care	Recovery-oriented	Yes^a^	Yes^b^
	Strengths-based	Yes	Yes
	Designated rehabilitation focus	Yes	Yes
	Voluntary engagement in rehabilitation	Yes^c^	Yes^c^
	Individualized care planning	Yes	Yes
	Transitional support	Yes^d^	Yes^d^
	Role of peer support	Minimal support	Minimal support
Treatment and support	Cognitive Behavior Therapy (CBT)	Yes	Yes
	Living skills support and development	Yes	Yes
	Structured leisure and physical activities	Yes	Yes
	Social integration and economic empowerment	Yes^f^	Yes^f^
	Evidence-based therapeutic group programs	Limited	Limited

^a^Recovery oriented approach (psychological rehabilitation, social integration and economic empowerment, eg cottage industry)

^b^Recovery oriented (assessment, detoxification, rehab treatment, addiction therapy, extended care)

^c^Consumers have voluntary engagement with rehabilitation activities but sometimes accepted through an involuntary approach

^d^Transitional support programmes at the residential facilities are limited

^f^Structured social integration and economic empowerment programmes are limited due to financial challenges

The Mental Health Authority (MHA) in Ghana is responsible for coordinating and managing mental health services. The mental health authority directly advises the Ministry of Health on mental health issues, particularly on policy formulation and implementation [47]. Also, the mental health authority is responsible for coordinating and planning mental health activities at the national, regional and district levels [47]. This notwithstanding, mental health services are faced with several challenges, which include limited funding sources, high unmet service needs, poor management of mental health cadres, low priority, irregular supply of drugs, limited services for marginalized groups, and poor state of psychiatric facilities [45]. Also, consumers of mental health services face individual-level barriers, such as negative attitudes, limited knowledge on services, high treatment cost as well as transportation and distance to mental health facility [45, 48, 56].

### Research approach

This study was conducted from June 2017 to May 2018. The study employed qualitative data collection method, involving the use of in-depth interviews and observation. The qualitative method allowed the researchers to interact with MHPs and consumers and listen to their subjective perspectives concerning recovery services and expectation towards recovery [57, 58]. The use of a qualitative method helped to generate contextually detailed and rich data that enabled an in-depth understanding of the topic [57, 59–62].

### Participants and sampling

The participants recruited for the study were consumers receiving mental health services and MHPs providing recovery services at community-based residential setting. The consumers and MHPs were jointly targeted because they have a shared goal and expectation towards recovery. Also, these participants were targeted because previous researches concentrated mostly on policy implementers in government agencies [47]. Little is known about the perspectives of MHPs and consumers on recovery services and expectation towards recovery using qualitative studies. We employed several steps to recruit participants. We extracted the background profile of consumers, who were admitted to the two community-based residential facilities. We purposively selected consumers who met the eligibility criteria. The inclusion criteria for consumers were that one must have been diagnosed by a MHPs or self-reported as having schizophrenia, substance abuse, depression, anxiety disorders, post-traumatic stress disorders and other addictive behaviours. Apart from the above, a prospective participant must have stayed in the facility for at least one month and must be cognitively stable (based on the most recent Mental Status Examination). Using the above information, we identified a pool of consumers who formed our sample frame. We invited these consumers through an email and phone call. The invitation package contained a letter, consent form and participants’ information sheet, which described the purpose of the research, selection process, the risk and benefits of participation, confidentiality and voluntary nature of participation. A total of 19 consumers, 20 years and above, from both facilities, were recruited. The consumers, who agreed to participate in the study provided suitable dates for the interview. This process was used to approach 10 MHPs, who provide daily routine recovery services to consumers in each of the community-based residential facility. However, five MHPs participants (occupational therapy, mental health nurses, prescriber/psychiatrist and social worker) agreed to participate in the interviews.

### Data collection

An in-depth interview and observation were used to collect data from all the participants. The data collection started with an observation, using an adopted checklist (Table 1 [17, 63] and followed by in-depth interviews. As part of a three-month field visit (June 2017 – August 2017) at the first facility, the lead author (N.G) commenced the initial data collection with an observation of all activities. The lead author (N.G) undertook three weeks visit to the second facility from November 2017 to perform a similar activity.

After the observation, the in-depth interviews started. The in-depth interviews allowed consumers and MHPs to give a detailed account of the recovery services and expectations towards recovery. Such in-depth information may not be disclosed by participants in a group level discussion [62]. The interviews were conducted using an interview guide, which was developed using previous literature and theory on recovery services (Additional file 1). The interview guide covered issues on recovery services (eg. service models, recovery interventions) and expectation towards recovery journey [1, 19, 20].

The interviews were conducted by two members (N.G and E.B) of the research team. All participants were briefed about the research objectives, procedures and the consent process. Participants were made to sign a written consent form before participation. The researchers sought permission to record all interview sessions. The in-depth interviews were conducted using an interview guide (Additional file 1). The interviewer read the questions on the interview guide to the participants and recorded the response, using an audio-tape recorder. All the in-depth interviews with consumers were conducted in Akan, the widely spoken local dialect in the study area. However, the MHPs’ interviews were conducted in the English language, the primary language of conversation in formal educational settings in Ghana. All the interviews were conducted at the staff common rooms, and each interview was conducted separately and privately, on the weekend; no interview was witnessed by a prospective participant. Each interview session took approximately 45 min to 1 h.

### Data analysis

The data were analyzed manually using thematic analysis adapted from Braun and Clarke [64]. The analysis involves transcribing, reading, familiarizing with the data, generating initial codes, searching for themes, reviewing themes, and rigorous interpretation of data. The researchers listened to the audio-recorded interviews back-to-back and reviewed the transcription and field notes for a fair understanding of the content of the transcripts.

The next step involved initial coding and focused coding to build categories as recommended by Saldaña [65]. Here, data from the interview with the consumer and MHPs were fragmented line-by-line and in segments, relinked to consolidate meaning and explanation. For example, in the focused coding, the most significant initial codes were used as provisional categories for checking with other interviews. The coding continued until saturation was reached –no new ideas emerged from successive coding. The meaning and relationships between codes were used to generate basic themes, organizing themes, and global themes (Table 3). The coding and categorization of basic, organizing and global themes were done in consultation with co-authors, who have years of experience in conducting qualitative research.

## Results

### Characteristics of participants

A total of 19 consumers and five MHPs participated in the study. More than half (57.89%) of consumers and 60% of MHPs were males (Table 2). More than a third of the consumers (42.10%) had SHS/Technical and Vocational education with more than a third (42.10%) being unemployed. Again, 57.89% of the consumer and 40% of the MHPs were single, whilst 26.31% of consumers and 60%of the MHPs were married. The average years of working experience for the MHPs was 3 years. Five participants, three MHPs and two consumers commented that the type of mental illness reported at the facilities were psychotic or severe conditions, such as schizophrenia, depression, bipolar and drug addiction. The participants also noted that the maximum treatment plan for consumers with addiction was 90 days (based on the standard protocol). However, there was an extension for consumers with dual diagnoses, such as psychosis and addiction.

**Table 2:** Background information of participants

*Variables*	*Facility 1*	*Facility 2*	*Total*
*Consumer participants*	N (%)	N (%)	N (%)
Gender			
Males	5 (55.55)	6 (60.00)	11 (57.89)
Female	4 (45.55)	4 (40.00)	8 (41.10)
Age			
20–30	4 (44.44)	–	4 (21.05)
31–40	1 (11.11)	4 (40.00)	4 (21.05)
41–50	2 (22.22)	2 (20.00)	2 (10.52)
51–60	–	2 (20.00)	2 (10.52)
61–70	2 (22.22)	2 (20.00)	2 (10.52)
Mean:40, SD:12			
Education			
Basic	2 (22.22)	4 (40.00)	6 (31.57)
SHS/technical and vocational	3 (33.33)	5 (50.00)	8 (42.10)
Tertiary	4 (44.44)	1 (10.00)	5 (26.31)
Marital status			
Single	6 (66.66)	5 (50.00)	11 (57.89)
Co-habitation	1 (11.11)	–	1 (5.26)
Married	2 (22.22)	3 (30.00)	5 (26.31)
Separated	–	1 (10.00)	1 (5.26)
Widow	–	1 (10.00)	1 (5.26)
Occupation			
Unemployment	2 (22.22)	6 (60.00)	8 (42.10)
Self-employed	3 (33.33)	2 (20.00)	5 (26.31)
Government sector	1 (11.11)	1 (10.00)	2 (10.52)
Student	2 (22.22)	–	2 (10.52)
Pastoral work	1 (11.11)	1 (10.00)	2 (10.52)
*MHPs*			
Profession			
Occupational therapist	1 (50)	1 (33.33)	2 (40)
Psychiatric nurses	1 (50)	–	1 (20)
Psychiatrist/Prescriber	–	1 (33.33)	1 (20)
Social worker		1 (33.33)	1 (20)
Gender			
Males	1 (50)	2 (66.67)	3 (60)
Females	1 (50)	1 (33.33)	2 (40)
Marital status			
Single	1 (50)	22 (66.67)	3 (60)
Married	1 (50)	1 (33.33)	2 (40)
Average years of working experience			3 years

### Themes identified from the thematic analysis

The study identified three themes and nine organizing themes. These themes were consistent across the responses by the MHPs and consumers (Table 3). The themes were also consistent with the findings from the observations. As illustrated in Table 1, the observation showed that the residential facilities operated using several principles that include recovery-oriented, strength-based approach, designated rehabilitation focus, voluntary engagement in rehabilitation, individualized care planning and transitional and peer support. The treatment and support provided at the facilities were cognitive behaviour therapy, independent living skills, structured leisure and physical activities, and social integration and therapeutic group programmes.

**Table 3:** Themes from the analysis

Global themes	Organizing themes	Basic themes	Codes
Theme 1: Recovery services offered to consumers	Admission process to the residential facilities	Conducting MSE and PHA are used to assess mental and physical health of consumers respectively	Mental Status Examination (MSE)
			Physical Health Assessment (PHA)
			Treatment plan and goals setting
		Receiving a referral from a psychiatric facility can be used to start recovery services	Continuity of services
	Routine recovery activities and intervention	Planning daily recovery activities is key in achieving recovery	Personal self-care skills
			Medication
			Time management skills
			Socialization (eg. playing games and watching television)
			Leisure and recreational activities
			Psycho-therapy
			Psycho-education
			Mindfulness-based interventions
			Physical health training
			Cognitive behaviour therapy
			Family therapy
	Consumers’ involvement in decision concerning recovery services	Respecting consumers rights in medication decision is key in achieving recovery	Consumers understand their rights
			Temporarily seizing consumers autonomy
Theme 2: Expectation regarding the personal recovery process	Psychiatric medication (management of condition)	Adherence to psychiatric medication improve the recovery process	Becoming sober
			Reducing aggression
			Improving meaningful conversation
			Reducing degree of illness
	Economic empowerment	Participating in normative life is key in recovery services	Expecting employment opportunities (eg. vocational training)
	Independent living	Attaining independent living	Regaining self-care and daily living skills
			Attracting respect
			Moving around independently
	Social inclusion (integration)	Gaining social inclusion	Integration into families and communities
			Making a meaningful contribution to families and society
			Participating in religious activities
			Social interaction (eg. entertainments)
Theme 3: Challenges in achieving personal recovery	Systemic or management-related challenges	Set-backs affecting implementation of recovery services	Limited funds
			Infrastructure
			Poor feeding
			Limited medication supply
			Limited family support
			Inadequate MHPs
	Consumer challenges	Consumers faced individual challenges	Dealing with unreasonable behaviour
			Uncooperativeness
			Non-adherence to medications

#### Theme 1: recovery services offered to consumers

This theme describes the various recovery services implemented and used to promote recovery for consumers. As shown in Table 3, the theme start by describing the process through which consumers are admitted into the community-based residential facilities. The theme also end by highlighting the routine recovery activities and interventions as well as the extent of consumers’ involvement in the decision concerning recovery services. The emerging issues are discussed below:

##### Admission process to the residential facilities

Both the MHPs and consumers mentioned two approaches through which consumers are admitted to the residential facilities. The consumers were admitted through initial assessment or referral from a psychiatric facility. For instance, all MHPs and two consumers indicated that the services were initiated through a Mental Status Examination (MSE) and Physical Health Assessment (PHA). The MSE of consumers was assessed to develop a treatment plan and recovery goals. In addition to this, most of the participants (both consumers and MHPs) noted that the PHA was conducted to identify any secondary co-morbid conditions:

*“The first thing is that you have to do Mental Status Examination to know what treatment plan you have for them. Secondly, when you draw the treatment plan you have to involve the family because they can give you the correct information on the consumer. So we first do the mental status examination and if the person has been to a different hospital, we write the medications down, and we also write if the family have a history of mental illness”* (MHP 4; Psychiatrist, Facility 2).

*“A nurse gave me treatment. The nurse tied my hand and took a sample of my blood [physical health assessment]. And they said only males are at where they took me. So I was brought here in the evening by car”* (Consumer 14, Facility 2).

*“When I arrived, they hold something which has been typed on paper. They looked on that to give me treatment. There was one nurse called Awurama [name]. She took care of me the first 3 months I came here n went back home”* (Consumer 13, Facility 2).

Additionally, some MHPs expressed that the recovery services were initiated through a referral from a psychiatric facility to provide a continuity of services. Most of the MHPs further expressed that family caregivers were needed to provide information on the clinical or medical history of consumers before the initiation of the services. A mental health nurse from facility 1, echoed on the admission process as follows:

*“Initiation comes from the first point of contact, which is the psychiatric hospital or facilities they were. When they come, they come and continue with their medication, except that the person is not responding to that treatment or we need to reduce it for their improvement”* (MHP 1; Mental Health Nurse, Facility 1).

Two MHP participants noted that apart from consumers with psychotic disorders, the initiation of recovery services for those with addiction started with counselling.

##### Routine recovery activities and interventions

Both the consumers and MHPs described the daily recovery activities and interventions. Most consumers (15/19 and MHP (3/5) said that after cleaning, bathing, breakfast, and morning devotion, the consumers took their medication and engaged in leisure and recreational activities, such as watching television (TV) and playing, each morning. In the afternoon, they continued with the same leisure and recreational activities. The responses from consumers and MHPs suggest that the daily activities were planned to improve their time management skills. Three consumers echoed on this:

*“When we wake up every day, we do household chores, like scrubbing the toilet and mopping the tiles. Afterwards, we, eat breakfast prepared by the caterers of the facility, eat lunch in the afternoon and evening supper, then sleep if you want to, if not you can rest or watch TV chat with others and take medicine”* (Consumer 16, Facility 2).

*“I brush my teeth, have my bath and ease myself. If the matron is not around, I read my magazine till the time the matron will call us to come for our food (porridge) … I will be awake at around 10 am and eat. I will go back to the room and if no one is disturbing I will lie down on the floor and exercise myself because I am a sportswoman”* (Consumer 13, Facility 2).

*“In the morning when we wake up, we bath and dress up after that we go for our meals and later the bell rings for us to take our medicine so, after the medicine, we are all sent to bed. Sometimes we don’t even sleep we just seat there idle till evening or lunch”* (Consumer 9, Facility 1).

Seven categories of recovery interventions were identified by all MHP participants (5/5). The recovery interventions identified were psycho-therapy, psycho-education, mindfulness-based interventions (conflict management, coping skills), physical health training, cognitive behaviour therapy, family therapy and leisure and recreational activities. The MHP stated that the psychotherapy, which is mostly provided to consumers with psychotic symptoms, was aimed at educating the consumers about the importance of the medication. The mindfulness-based strategies, which were mostly provided to consumers with drug addiction, was aimed at educating them on how to manage conflicts, particularly when discharged back into the community. An occupational therapist commented on the recovery interventions as follows:

*“Every Monday we do group psychotherapy. We try to educate the psychotic patient. For the addicts, on Tuesdays, we choose one particular disease or a condition like malaria and talk about it. So after their breakfast, we gather them and talk. Sometimes we put them into groups and we share amongst ourselves. Also, for the drug addict, we do conflict management and coping skills for them. We have so many programs that we do”* (MHP 3; Occupational Therapist, Facility 2).

Some MHPs and consumers narrated that cognitive behaviour therapy was provided to change the mindset and believes of consumers, particularly regarding the definition of mental illness:

*“The cognitive behaviour therapy is to change the mindset. We believe if you change your thinking, it should change your behaviour., we have a deeper task which is one on one to look at things that have influenced you and your believes about taking the drugs or something and then we try and help you reconstruct it”* (MHP 1; Mental Health Nurse, Facility 1).

Six consumers narrated the availability and their participation in recovery services, including leisure and recreational activities, (eg. playing of games and exercise) and psychoeducation (eg. health education and training). For instance, consumer 10 from facility 1 stated that *“there are games here such as ludo [leisure and recreational activity] so if you are not interested you go to bed”.* Consumer 13 from facility 2 also mentioned that “*I will lie down on the floor and exercise myself because I am a sportswoman*”. Moreover, another consumer from facility 2, commented on the psychoeducation as “*we do some health training [psychoeducation] after that we eat then we sleep. Then we take our medicine.*

The narrations from consumers confirm the response from MHPs concerning the existing recovery services provided to consumers. Despite these recovery services, another MHP added that due to inadequate funding, they were unable to provide adequate occupational therapy and vocational training to fully reintegrate the consumers into the community:

*“Apart from the normal routine activities, bathing, prayers, playing games, taking their medication, watching TV and having leisure time, we wanted to add occupation therapy and vocational training to fully reintegrate them into the community. But that has not been successful due to inadequate funds”* (MHP 1; Mental Health Nurse, Facility 1).

*Consumers’ involvement in the decision concerning recovery services*

The consumers and MHPs shared their perspectives regarding the extent of consumers’ involvement in the decision in recovery services. The MHPs narrated that consumers, particularly those with addiction issues, who have insights into their conditions, were mostly involved in the medication plan. A social worker commented on the consumers’ involvement as follows:

*“Most of them have insight so they are involved … Some of them know [the reactions of the medicine] because they have insight. Some of them also do not have insight so you have to put them on the start and then see their way forward”* (MHP 5; Social Worker, Facility 2).

The MHPs felt that respecting consumers’ rights and involvement in decision concerning their lives are key in achieving personal recovery. These perspectives align with the principles of recovery-oriented practices, which support consensus in developing recovery goals and plan.

The perspectives of MHPs regarding the involvement of consumers in the recovery decision were also consistent with the response from a section of consumers (5/19). The consumers indicated that they were allowed to make choices and decisions regarding recovery services. For example, consumer 18 from facility 2 stated that “*Yes, if I want to take decisions, I sit down and I plan my things … mmm”.* Other consumers also gave instances where they have contributed to the service plan, particularly on the choice of services. In particular, consumer 9 from facility 1 stated that “*Yes, there is a medicine that makes me feel dizzy so when I told them, they changed it and gave me another medicine”.*

All the MHPs also noted that the Mental Health Act 842, (2012) gives the consumers the right to decide on medication. However, some MHPs mentioned that the Mental Health Act 842, (2012) permits MHPs to temporarily seize the autonomy of consumers, particularly those who are psychotic and aggressive, to prevent them from causing harm to themselves and others. For instance, an occupational therapist echoed that some consumers who were psychotic have no insight to make any decision:

*“ … a patient who has psychosis for the first time has no insight so he cannot make any decision. The law put it in this way that, at that particular moment, decision is going to be made for him. We decide because they can cause injury to themselves and others. So to take them out of the danger, their autonomy has to be seized temporary”* (MHP 3; Occupational Therapist, Facility 2).

#### Expectation regarding the personal recovery process

This theme explains the shared expectations of MHPs and consumers regarding the recovery journey of consumers. This section is explained according to four organizing themes, including adherence to psychiatric medication, economic empowerment, independent living and social inclusion. The sub-themes emerging are explained in the following sections:

##### Adherence to psychiatric medication

The narratives from the majority of MHPs and consumers demonstrated that adhering to psychiatric medication was very important towards the recovery of consumers. The psychiatric medications help consumers to be safe and sound in mind, sober, less aggressive and to undertake meaningful conversation. Although these attributes are similar to the clinical recovery model, they are also important part of illness management in achieving personal recovery of consumers. For instance, a social worker expressed that though they believed in personal recovery, adherence to psychiatric medication helps to improve the process:

*“It’s helpful because without the medication we cannot stay with them (achieve personal recovery). We live with them based on the treatment. We believe that when they take medication, they become sober so we believe in the medication”* (MHP 5; Social Worker, Facility 2).

Similarly, most of the consumers agreed that psychiatric medication did not only reduce the symptoms, but increased their level of functioning, and this appears to be the motivation to adhere to psychiatric medication – it, enabled them to feel better and engage in meaningful conversations. For instance, two consumer participants described the importance of adhering to the psychiatric medication in the remarks below:

*“After the patient [we] is given medication, about one month, you would realize that the degree of the illness has reduced to the extent that you can have a meaningful conversation with him and he can do everything properly”* (Consumer 4, Facility 1).

*“As I am taking my treatment, to the glory of God, I will be better and well treated. I will be going home. And after that, I will be coming here for my treatment and when I go home I can start working. It will help me to abstain from wee [marijuana] smoking”* (Consumer 2, Facility 2).

##### Economic empowerment

Most of the consumers (15/19) explained that their expectation was to be empowered economically by the time they were discharged from the facilities. This theme is based on the notion that community-based residential facilities can give them some vocational training skills to take up employment opportunities. The consumers’ expectations regarding vocational activities and employment opportunities could help attain economic empowerment. Three categories of employment opportunities were identified by the consumers. These included artisanship, petty trading (selling tomatoes and pepper and distribution of antibiotics) and agricultural activities (growing maize). Three consumers echoed their expectations to participate in income generation activities when they returned home:

*“Yes to go home and help them plant the maize because a season like June, July, August and September is very good at planting foodstuffs as compared to other months.* (Consumer 11, Facility 2).

*“Well, when I leave this place, I know how to sew clothes. I know how to sew clothes so when I leave this place, my aim is either to travel [go abroad] or be able to set up a shop where I would be able to work”* (Consumer 4, Facility 1)

*“I want to get well so I will go home and work … I want to be a hairdresser. I don’t know how to fix the wig but I can do other things”* (Consumer 9, Facility 1)

Despite the above expectations, our analysis highlighted that resource constraints within the service make it challenging to provide sufficient economic empowerment activities that could support consumers to be more competitive in the market. For example, a MHP recounted that several attempts have been made to use the vocational training to empower the consumers economically, but they were not able to compete with other products in the market because theirs were inferior compared to other products, and were sold at lower prices. This challenge is captured in the quotation below:

*“They use to do a lot of things but the turnover was poor because people [consumers] will produce a lower [substandard] product and they will sell at a lower cost. Liquid soap, for instance, the cost [market price] was around GHC 1[less than $1] but the people were selling it at the market place for 50p. So we were running at a loss, unless we get some support we can’t start again or invest in that vocational activity”* (MHP 1; Mental Health Nurse, Facility 1).

##### Independent living

Most of the MHPs (4/5) and consumers were of the view that the recovery service offered to consumers was effective to help them acquire independent living skills. Some MHP explained that most consumers were expected to attain independent living in areas such as daily living skills and self-care management. They expected that consumers who passed through the recovery services would be able to successfully manage themselves. For example, a social worker noted that consumers were able to undertake daily self-care activities:

*“We expect that they can work for themselves and they will never depend on somebody. We are expecting that, after the rehabilitation, they can use the things that they learn from here to go out there to manage their lives without depending on somebody”* (MHP 5; Social Worker, Facility 2).

Some MHPs and consumers mentioned that attaining independent life would help consumers to be respected, remain calm, and move around on their own. For instance, one consumer stated that “*I see myself after rehabilitation as going to the house alone, calm and respectful.*” Another consumer confirmed this assertion in the quote that follows:

*“What I came here to seek … I have achieved it. I can go outside. I go to church at Grace Baptist every Sunday. Every Sunday I take a car from this place to Grace Baptist. So even on the days when I do not go anywhere, I can go out and take a walk. If I was still smoking the wee [marijuana], would I have been able to do all this? That’s why I was saying that, as I stay here, all that I wanted to achieve. I have achieved”* (Consumer 4, Facility 1).

##### Social inclusion

Social inclusion was identified as one of the major expectation of both MHPs and consumers. The social inclusion encompasses the ability of the consumers to participate fully in normative activities, such as education, health, social, political, cultural and religion. Social inclusion also entails removing stigma and discrimination perpetrated against consumers in their immediate environment and making them feel part of society. All MHPs and some consumer expressed that they expected consumers to be integrated into their communities and families when they are discharged. Some participants added that they expected the consumers to contribute meaningfully to their families, environment, and society at large when they are discharged.

*“That people will go to their home safe and sound and can interact with their family members normally, and participate in social life”* (MHP 1; Mental Health Nurse, Facility 1).

Some recovery services that could promote social inclusion and integration identified from the themes were community visits and participation in religious activities (eg. church). For instance, some MHPs said that they usually embark on follow-up visits to the homes and workplaces of consumers, who have been discharged, as exemplified in the following quote by a social worker:

*“In their community, we do a community visit, so we go to them … I see them in their community often and also those who are working, we go to their workplaces. Some of them were working before their condition, so we try and go to their workplaces and see how they are working”* (MHP 5; Social Worker, Facility 2).

Further, most MHP and consumer considered the residential facilities as a place of socialization that could promote recovery. According to both participants, the social activities, help the consumers to learn how to live with others, and, thus, helped to integrate them into their respective communities:

*“Apart from the medications to give them sound mind, recreational therapy has an impact on their socialization. When they play the game together, they love each other and are happy so relating with others in the society will not be a big issue anymore”* (MHP 1; Mental Health Nurse, Facility 1).

#### Challenges in achieving personal recovery

This theme highlights the challenges confronting the implementation as well as accessing the recovery services. The theme is discussed according to two sub-themes, which include the systematic, and management challenges as well as consumer-related challenges in terms of developing skills to manage and live with symptoms. The sub-themes are as follows:

##### Systemic and management related challenges

The MHPs identified some systemic related challenges that affected the recovery services. The system-related challenges identified by the MHPs were limited funds, inadequate infrastructure, poor feeding, limited medical supply, limited family support, and inadequate MHPs. For instance, most of the MHPs (4/5) said the facilities had limited funds to provide adequate feeding, electricity as well as medicine:

*“The major problems we have are feeding and drugs. Because our perception [about mental illness] is wrong, people do not even know they have to support their wards [consumers]. When they [family carers] bring them, they will never come back. We cannot live with them without giving them medication, so we use the little money we have to buy them medicine”* (MHP 5; Social Worker, Facility 2).

The responses from some of the consumers were consistent with those expressed by the MHPs. For instance, some consumers noted that there were several challenges: poor lighting, inadequate infrastructure and psychiatric medicines. These challenges affected the psychological well-being of the consumers. For example, some of the consumers said the poor lighting system adversely affected their sleep. Two consumer-described the challenges as follows:

*“Here the feeding is poor, where we sleep is also not comfortable. If you should go to my room, I am a neat person, so I can keep it tidy. But if you should go to some of the places where the other patients sleep, there isn’t even light there, bedbugs … and when we talk about feeding, we can eat some kinds of rice”* (Consumer 4, Facility 1)

*“They don’t have that much money to cook foods as you would have at home. They can’t make it for you as you would have it at home”* (Consumer 4, Facility 1)

##### Consumer-related challenges

Participants (MHPs and consumers) highlighted some resource challenges that affect consumers in terms of developing skills to manage and live with symptoms. For example, all MHPs narrated that the challenges were associated with dealing with behaviour changes, uncooperative attitude and noncompliance. Most MHPs mentioned that some of the consumers sometimes did not comply with the service plan. Also, the behaviour of some consumers delayed the recovery process*.*

Three consumers also recounted that they sometimes find it difficult to sleep especially being lonely and routinely doing the same thing. These challenges expressed by consumers seems to influence the behaviour changes expressed by the MHPs. For example, Consumer 19 from facility 2, stated that *“the challenges that I am facing is about sleeping. I do not sleep well … Yes, I do not sleep well*”. In addition, consumer 12 from facility 2, shared the same sentiment as “sometimes I can’t sleep in the afternoon”. Moreover, consumer 10 from facility 1 confirmed that they are unable to sleep, especially when their medication finishes. The consumer echoed as “*Yes and it’s about my medicine sometimes when my medicine gets finished if I don’t get them, I don’t sleep*”.

## Discussion

The study findings have answered the research question on the perspectives of MHPs and consumers concerning the recovery services as well as their expectation towards the recovery journey. The findings of the study were discussed using three organizing themes 1) recovery services offered to consumers 2) expectation regarding the recovery and 3) challenges in achieving personal recovery.

### Recovery services offered to consumers

The findings of the study showed that consumers were admitted into the facilities through assessments or referrals from psychiatric facilities. The assessment was based on MSE and PHA. Consistent with previous studies [24–30], the empirical findings from interviews and observations highlighted that the residential facilities implemented several recovery services through illness management, psychotherapy, psycho-education, mindfulness-based interventions, physical health training, cognitive behaviour and family therapy as well as leisure and recreational activities. The services were valuable in helping consumers to achieve personal recovery. The cognitive behaviour therapy, for example, is meant to change the mindset of consumers regarding the perfection of mental illness. Further, the psychotherapy and mindfulness-based strategies are relevant to educate consumers on how to manage or mitigate conflicts, which is necessary for community integration. Although the residential facilities were implementing some recovery services, there was limited application of evidence-based interventions. For instance, the recovery services tend to focus more on integrated treatment approaches (eg. Illness management), with relatively little attention on vocational rehabilitation (eg. Individual Placement Services) [34–36] as well as recovery photovoice, art-making and exhibition interventions. Past studies have argued that vocational rehabilitation, together with recovery photovoice, art making and exhibition services, are equally valuable interventions that can support the personal recovery of consumers [24–30]. For instance, a study by Drake and Whitley [1] suggested that supported employment and housing services can promote the recovery of consumers. It is therefore important for policymakers and MHPs to prioritize and incorporate this component of recovery interventions into the services.

### Expectation regarding the recovery process

The expectation of consumers and MHPs regarding the personal recovery process is relevant to transform consumers [2]. The study findings demonstrated that the recovery services were expected to effectively transform the internal recovery process, particularly helping consumers to attain independent living in areas, such as self-care and daily living skills. Recovery services are relevant in equipping consumers to become independent in managing their self-care and daily living activities [10, 12]. The expectation regarding the internal recovery process in the current study is consistent with previous studies that found that recovery services can build the inner strength and willpower of consumers [10, 12, 21]. This could help consumers to learn about their self-concept, understand the illness, change their lifestyles and reclaim various aspects of self. Particularly, the internal process can strengthen the self-confidence of consumers to take meaningful steps towards their self-care and wellbeing [3, 12, 13, 21]. More so, the internal process could also involve re-building a positive sense of identity in the face of illness [66]. The findings, thus, encourage MHPs to promote the internal recovery process of consumers, which can be achieved by effectively motivating consumers to take active roles in the recovery process [16].

Also, recovery services are expected to achieve an external recovery process of consumers. Accordingly, the services are expected to promote recovery processes in areas such as managing illness, enhancing economic empowerment, and promoting social inclusion or community integration. Consistent with past studies [67, 68], MHPs and consumers expected the recovery services to help reduce aggressiveness and manage the illness, especially to reduce the symptoms. The expectation that consumers could feel better after receiving recovery services is consistent with previous studies that measure the effectiveness of mental health services on consumers [67–69]. In addition, recovery services are expected to economically empower consumers. Consumers are expected to participate in vocational training and employment opportunities when they return to the community. Participation in vocational activities in the facilities is relevant to helping consumers achieve economic independence to contribute meaningfully to the community. The findings corroborate with other studies that identified that vocational training and employment opportunities can help consumers to participate in livelihood and income-generating activities [26, 28]. In some instances, these activities helped consumers to gain financial literacy skills (eg. managing finances) and become financially stable [26, 28]. MHPs are encouraged to embark on vocational rehabilitation programmes, such as individual placement services, to build the capacity of consumers. The participation of consumers in these activities in the residential facilities can empower them economically before being discharged into the community.

Both the consumers and MHPs expected recovery services to improve the social inclusion and community acceptance of consumers. The recovery services are expected to integrate consumers into their families and communities and, subsequently, contribute to society. The community integration can be achieved through effective participation in religious activities, festivals and community gathering [1, 3, 10, 16]. The expectation regarding social inclusion confirms previous studies on the effectiveness of recovery services [1, 3, 9–13, 16, 17]. Consumers can achieve social inclusion and integration through participation in recreational activities (socialization), social connectedness, and social interaction [1, 3, 9–17]. The social inclusion activities can help to reduce discrimination, social isolation, increasing social contacts and creating a supportive social environment. The findings recommend that the current social inclusion strategies used by the residential facilities should be strengthened and monitored to improve the external recovery process of consumers.

### Challenges in achieving personal recovery for consumers

The study identified several systemic as well as consumer-related challenges that hindered the effective attainment of the personal recovery process. The systematic related challenges were related to limited funds, poor infrastructure support, poor feeding, limited supply of drugs and inadequate family support. The limited financial support affects the implementation of vocational rehabilitation programmes that can facilitate effective community integration and economic empowerment of consumers. These challenges are caused by limited priority and support from government, families and communities towards mental health services. In Ghana, there are several weaknesses in the mental health systems, due to the competing development priority. Consequently, mental health services have historically received limited attention from the government, particularly in areas such as funding, policies, infrastructure support and human resources development [45–48]. Mental health systems, occasionally, receive support from external sources, which is relatively not sustainable and inadequate to meet the increasing needs of services. The limited supply of MHPs and the lack of training in recovery can hinder the effective achievement of personal recovery goals of consumers. The systemic and management related challenges mostly affect the effective implementation of recovery services. We recommend that government should prioritize and support recovery services that are geared towards the personal goals of consumers. Moreover, consumer-related challenges such as the difficulty in dealing with behaviour changes and non-adherence to illness management plans affected the implementation of recovery services. The consumers of services, particularly those with psychosis often exhibit aggressive behaviour. In some instances, MHPs, who lack the relevant skills in managing aggressive behaviours find it difficult to handle such consumers. Given this, MHPs should be given in-service training to improve their knowledge regarding recovery and how to handle aggressive consumers. This is because consumer related challenges can negatively impact on personal recovery process [1, 12, 44].

### Limitations

The study has several limitations that need consideration. The limitations are associated with the scope that is, recruiting two residential facilities, data collection instruments, sampling and vulnerability of participants. The study was limited to only MHPs and consumers of mental health services in two community-based residential facilities, without the perspectives of mental health policy planners from government ministries. The interview guide used for the data collection was developed by the researchers without adopting a validated data collection instrument. Also, the study employed purposive sampling to recruit participants who had some characteristics. Again, consumers probably withheld vital information considered sensitive to them or socially inappropriate. These limitations have the potential to affect the trustworthiness of the findings. However, the researchers used several methodological rigour to ensure the trustworthiness of the results. We adhered to the principles of credibility, confirmability, transferability, dependability and reflexive reporting. We piloted the interview guide before the actual fieldwork. The thematic analysis process used to analyze the findings was subjected to coding by consensus, member checking, and a series of debriefing sessions. Finally, the findings have been discussed with previous literature on recovery services.

## Conclusion

This paper explored the perspectives of consumers and MHPs regarding recovery services and their expectation towards recovery. The study identified several recovery services that were being provided to consumers. These services include psychotherapy, psycho-education, mindfulness-based interventions, physical health education, cognitive behaviour and family therapy as well as leisure and recreational activities. The study concludes that recovery services are expected to effectively transform the internal and external recovery process of consumers. The internal recovery process was independent living in areas such as self-care and daily living skills. Similarly, the external recovery process was illness management, economic empowerment, and social inclusion or community integration. Despite these expectations, several systemic and consumer-related challenges hindered the personal recovery journey. The systemic related challenges were limited funds, poor infrastructure support, poor feeding, limited medication supply, limited family support and MHPs. Conversely, the consumer-related challenges that affected consumers and MHPs expectation towards recovery were the difficulties in dealing with unreasonable behaviour, uncooperativeness and non-adherence to medications.

### The implication for policy and mental health nursing practice

The paper aims to explore MHPs and consumers perspectives on the implantation of recovery services and expectation towards recovery. The findings from the study are relevant to inform policy, mental health nursing practices, advocacy as well as the education of MHPs. Firstly, based on the findings, we recommend that the current recovery services should be monitored and evaluated to effectively achieve personal recovery journey. Secondly, the existing recovery services should incorporate vocational rehabilitation interventions such as sheltered and supported employment opportunities to improve the economic empowerment and personal recovery process. Thirdly, government policy towards mental health services should prioritize recovery services. Importantly, the government should provide adequate financial incentives, infrastructure support, and in-service training to MHPs to abreast their knowledge on how to implement recovery services. Also, the government should collaborate with MHPs, consumer self-help groups, family caregivers and other mental health stakeholders to embark on advocacy campaigns in the communities to increase community acceptance, reduced stigma and discrimination, particularly when consumers are discharged into the communities. Lastly, future research should use interventional studies to measure the effectiveness of recovery services on the lives of consumers discharged into the communities.

## Supplementary information

**Additional file 1.** Questions covered in the interview guide.

## Data Availability

All data supporting these findings are either contained in the manuscript or available upon request. There are no restrictions to anonymized data sources. All data collection tools, including interview guides and information and consent forms, are also available upon request.

## Abbreviations

CHRPE: Committee on Human Research and Publication Ethics
IDIs: In-depth interviews
KNUST: Kwame Nkrumah University of Science and Technology
MHA: Mental Health Authority
MHPs: Mental Health Professionals
MSE: Mental Status Examination
PHA: Physical Health Assessment
PHC: Primary Health Care
TV: Television
