# Supplementary material for: Recovery services and expectation of consumers and mental health professionals in community-based residential facilities of Ghana
Source: BMC Psychiatry. 2020 Jul 6;20:355. doi: 10.1186/s12888-020-02768-w (PMC7339466; doi:10.1186/s12888-020-02768-w)
Supplement: Supplementary file 1 — Additional file 1. Questions covered in the interview guide. [file 12888_2020_2768_MOESM1_ESM.docx]

Additional file 1: Questions covered in the interview guide

| Participants | Questions |
| --- | --- |
| MHPs | 1. What category of consumers receives recovery services at this residential facility? 2. What is the process involved in the initiation of recovery services for consumers? 3. What are the daily routine activities at this residential facility? 4. What specific role do you play in managing the routine activities daily? 5. What are the available recovery services provided to consumers? 6. How do specific interventions contribute to the personal recovery journey of consumers? 7. Tell me about how you involve the consumers and their relatives in the rehabilitation process? 8. Tell me about your expectation from the recovery services provided at this residential facility? 9. How does this expectation help to attain personal recovery for the consumers? 10. How does your role contribute to achieving the expectation regarding the personal recovery of consumers 11. What are the challenges confronting the attainment of the personal recovery goals of consumers? |
| Consumers | 1. What category of consumers receives recovery services at this residential facility? 2. Tell me about how you were admitted into this residential facility? 3. Tell me about the daily routine activities at this residential facility? 4. How do you adhere to the daily routine activities at this residential facility? 5. What is the range of recovery services that are provided in this residential facility? 6. Tell me about how the specific rehabilitation services support your personal recovery journey? 7. Tell me about the extent to which your close relatives are involved in the rehabilitation process? 8. What is your expectation from the recovery services you are receiving from this residential facility? 9. Where do you expect to go when you are discharged from this residential facility? 10. How can the specific rehabilitation services help you to achieve this target? 11. What are the specific challenges you faced from the recovery services provided at this residential facility? |
